# Different Subsets of Macrophages in Patients with New Onset Tuberculous Pleural Effusion

**DOI:** 10.1371/journal.pone.0088343

**Published:** 2014-02-10

**Authors:** Ying Tang, Shu-Cheng Hua, Gui-Xiang Qin, Li-Jun Xu, Yan-Fang Jiang

**Affiliations:** 1 Department of Respiratory Medicine, The First Hospital, Jilin University, Changchun, China; 2 The Center of Tuberculous Meningitis Diagnosis and Treatment, The Infectious Disease Hospital of Changchun, Changchun, China; 3 Key Laboratory for Zoonosis Research, Ministry of Education; Department of Central Laboratory, The First Hospital, Jilin University, Changchun, China; University of Cape Town, South Africa

## Abstract

**Objective:**

Macrophages are the infiltrate components of tuberculous pleural effusion (TPE). This study is aimed at examining the role of different subsets of macrophages in pleural fluid (PF) and peripheral blood (PB) from patients with new onset TPE.

**Methods:**

The numbers of PB and PF CD163^+^, CD206^+^ and CD115^+^ macrophages in 25 patients with new onset TPE and 17 healthy controls (HC) were determined by flow cytometry. The concentrations of serum and PF cytokines were determined by cytometric bead array (CBA) and enzyme-linked immunosorbentassay (ELISA). The potential association between the numbers of different subsets of macrophages and the values of clinical measures in TPE patients were analyzed.

**Results:**

The numbers of PB CD14^+^CD163^−^ M1-like and CD14^+^CD163^−^ interleukin (IL)-12^+^ M1 macrophages were significantly higher than that in the HC, but lower than PF, and the numbers of PF CD14^+^CD163^+^, CD14^+^CD163^+^CD206^+^, CD14^+^CD163^+^CDll5^+^ M2-like, and CD14^+^CD163^+^IL-10^+^ M2 macrophages were less than PB in the TPE patients. The levels of serum IL-1, IL-6, IL-8, IL-12, tumor growth factor (TGF)-β1, and tumor necrosis factor (TNF)-α in the TPE patients were significantly higher than that in the HC, but lower than that in the PF. The levels of PF IL-10 were significantly higher than that in the PB of patients and HC. In addition, the levels of serum IL-12 and TNF-α were correlated positively with the values of erythrocyte sedimentation rate (ESR) and the numbers of ESAT-6- and culture filtrate protein 10 (CFP-10)-specific IFN-γ-secreting T cells, and the levels of PF TNF-α were correlated positively with the levels of PF adenosine deaminase (ADA) and lactate dehydrogenase (LDH) in those patients.

**Conclusion:**

Our data indicate that *Mycobacterium* tuberculosis (M. tb) infection induces M1 predominant pro-inflammatory responses, contributing to the development of TPE in humans.

## Introduction

Tuberculous pleural effusion (TPE), a form of extra-pulmonary tuberculosis (EPTB), occurs in approximately 5% of patients with *Mycobacterium* tuberculosis (M. tb) infection [1]–[3]. Increased evidence indicates that following infection, M. tb induces innate immune responses [4]–[6]. Macrophages, as a type of antigen-presenting cells (APC) in the innate immune system, can efficiently present antigen to T cells in tissue, which results in T cell activation [7]–[8]. In addition, macrophages can produce numerous types of inflammatory mediators, such as IL-1β, TNF-α, IL-12, reactive oxygen species (ROS), and reactive nitrogen species (RNS), such as nitric oxide (NO), against *Mycobacteria* [9]. Macrophages are infiltrating components in the pleural fluid (PF) of TPE patients. However, little is known about whether the numbers of macrophages in the PF are similar to that in the peripheral blood (PB) of patients with new onset TPE and how they are different from that in healthy controls (HC). Hence, illustration of the change in the numbers of macrophages may help in understanding the role of macrophages in the early pathogenic process of TPE.

Monocytes and macrophages are heterogeneous cell populations. Blood monocytes can maturate into macrophages [10]. Macrophages acquire distinct functional phenotypes, depending on the microenvironment, and macrophages can be classically activated as M1 or alternatively activated as M2 [11]. Macrophages express CD14, a co-receptor of TLR4, which has been commonly used for identifying monocytes and macrophages. CD163 is a classical marker of M2 macrophages, while there is no reliable surface marker for the identification of M1 cells [12]. CD206 and CD115 are expressed by activated M2 and modulate the function of M2 [13]. Following activation, M1 macrophages secrete interleukin (IL)-12, which induces Th1 immune response, while M2 cells predominantly produce anti-inflammatory cytokines, such as IL-10 [11]. These different subsets of macrophages collaborate to control the overall inflammatory response. However, how these different subsets of macrophages exist in the PB and PF of TPE patients has not been clarified.

M. tb infection induces potent T cell responses and promotes T cell proliferation, which require high activation of adenosine deaminase (ADA) to support purine metabolism. Immunoreactivity-related tissue damage also increases the levels of lactate dehydrogenase (LDH), and inflammation also increases the erythrocyte sedimentation rate (ESR). Indeed, the levels of PF ADA and LDH and the values of ESR have been used for the diagnosis of TPE in the clinic [14]–[17]. However, how the numbers of different subsets of macrophages are related to the values of clinical measures has not been systemically investigated.

In the current study, we examined the numbers of PB and PF monocytes/macrophages in patients with new onset TPE, and we analyzed the potential association of the numbers of different subsets of monocytes/macrophages with the values of laboratory measures in the TPE patients. We found significantly increased numbers of M1 cells, but decreased numbers of M2 cells in the patients, particularly in the PF. Similarly, significantly elevated levels of pro-inflammatory cytokines were detected in the patients, especially in the PF. Our data support the notion that M. tb infection induces a polarized M1 response, which may participate in the early pathogenic process of TPE in humans.

## Materials and Methods

### Subjects

A total of 25 patients with new onset TPE were recruited from the inpatient service of the First Hospital of Jilin University, Changchun, China. Individual TPE patients with clinical symptoms <30 days were diagnosed based on the evidence of *M*. tb on a pleural effusion smear, growth of *M*.tb from pleural effusion or positive pleural biopsy specimen with granulomatous pleurisy [3]. All of the patients had no history of pulmonary tuberculosis and did not receive any treatment with anti-tb, steroids and immunosuppressants before their specimens were collected. Individual patients were excluded if she/he had concomitant conditions, including autoimmune disease, human immunodeificiency virus (HIV) infection, cancer, pregnancy, extra-pulmonary TB, and another systemic disease. Another 17 gender-, age-, and ethnic-matched healthy subjects were also recruited at the Physical Examination Center of the same hospital and served as healthy controls (HC). Written informed consent was obtained from individual participants. The experimental protocol was established, according to the guidelines of the 1975 Declaration of Helsinki and approved by the Human Ethics Committee of Jilin University, China.

### Clinical examination

The clinical data of each subject were collected from the hospital records. These data included age, sex, and laboratory tests. Individual subjects were subjected to routine laboratory tests for full blood cell counts. The levels of PF LDH, ADA, and carcinoembryonic antigen (CEA) were detected by the enzyme colorimetric assay and chemiluminescence immunoassay using special kits, according the manufacturers’ instructions (Huachenbio, Shanghai, China), (Aosibangbio, Yantai, China), and (Beckman Coulter, Fullerton, CA, USA), respectively.

### Flow cytometry analysis

PB mononuclear cells (PBMCs) and PF mononuclear cells (PFMCs) were isolated from individual subjects by density-gradient centrifugation using Ficoll-Paque Plus (Amersham Biosciences, Little Chalfont, UK). To determine the numbers of different subsets of monocytes/macrophages, PBMCs (10^6^ cells/tube) or PFMCs (10^6^ cells/tube) were stained with FITC-conjugated anti-CD206, PE-conjugated anti-CD115, peridinin-chlorophyll-protein complex (PerCP)-conjugated anti-CD14, and Alexaflour-647-conjugated anti-CD163 (BD PharMingen, San Diego, USA). The matched mouse isotype controls included FITC-IgG1, PE-Ig2a, PerCP-IgG1, and Alexaflour-IgG1. After being washed with phosphate buffered saline (PBS), the cells were characterized by flow cytometry using a FACS Calibur (BD Biosciences, San Jose, USA) and FlowJo software (v5.7.2) (TreeStar, Ashland, OR, USA). Because of the lack of reliable M1 markers, we considered CD14^+^CD163^+^ cells as M2-like cells, while CD14^+^CD163^−^ cells as M1-like cells.

To detect the function, PBMCs or PFMCs (10^6^ cells/well) were stimulated in duplicate with 50 ng/ml of lipopolysaccharide (LPS) and phorbol myristate acetate (PMA) and 1.0 µg/ml of ionomycin (Sigma-Aldrich, St. Louis, USA) in complete RPMI 1640 medium for 2 hours at 37°C in 5% CO_2_ and exposed to Brefeldin A (GolgiPlug; BD Biosciences) for 4 hours. After being washed, the cells were stained with PerCP-anti-CD14 and Alexaflour-647-anti-CD163 (BD PharMingen), fixed, and permeabilized using the permeabilization solution (BD Biosciences), followed by intracellular staining with FITC-anti-IL10 and PE-anti-IL-12. The percentages of IL-10^+^ and IL-12^+^ monocytes/macrophages were determined by flow cytometry. The numbers of each type of monocytes/macrophages were calculated, according to the total WBC and monocytes/macrophages. Because there were almost no detectable IL-10^+^ M1-like and IL-12^+^ M2-like cells, we defined the IL-12^+^ M1-like cells as M1 and IL-10^+^ M2-like cells as M2.

### Cytometric bead array (CBA) for the levels of serum and PF cytokines

The concentrations of serum TNF-α, IL-1β, IL-6, IL-8, IL-10, and IL-12 were determined by CBA, according to the manufacturer’s protocol (BD Biosciences) with minor modification. Briefly, individual serum samples (50 µL) were subjected in duplicate to analysis of the levels of serum and PF TNF-α, IL-1β, IL-6, IL-8, IL-10, and IL-12 in individual subjects using the CBA kit on a FACS Calibur cytometry. The concentrations of serum and PF cytokines were quantified using the CellQuestPro and CBA software (Becton Dickinson). The limitation of detection for TNF-α, IL-1β, IL-6, IL-8, IL-10, and IL-12 is 3.7 pg/ml, 7.2 pg/ml, 2.5 pg/ml, 3.6 pg/ml, 3.3 pg/ml, and 1.9 pg/ml, respectively.

### Measurement of serum and PF TGF-β1 by enzyme-linked immunosorbent assay (ELISA)

The concentrations of serum and PF bioactive TGF-β1 in individual subjects were determined by ELISA using a human TGF-β1 ELISA Kit, according to the manufacturers’ instruction (R&D Systems, Minneapolis, MN, USA). Briefly, individual sera at 1:4 dilutions were subjected to ELISA analysis in triplicate, and the concentrations of serum and PF TGF-β1 in individual samples were calculated, according to the standard curve established using the recombinant TGF-β1 provided. The limitation of detection for TGF-β1 is 15.4 pg/ml.

### T-SPOT.TB

The numbers of antigen-specific IFN-γ-secreting T cells in individual subjects was determined by the T-SPOT.TB assay using a commercial kit, according the manufacturers instruction (Oxford Immunotec, Oxford, United Kingdom). Briefly, 5 mL of venous blood were collected from individual subjects and immediately subjected to analysis in duplicate. The numbers of spot forming cells (SFC) were counted by two experienced scientists in a blinded manner.

### Statistical analysis

Data are expressed as median and range unless specified. The difference between groups was analyzed by the Kruskal-Wallis test or Chi-square test using the SPSS 16.0 software. The relationship between variables was evaluated using the Spearman rank correlation test. A two-side P value < 0.05 was considered statistically significant.

## Results

### Demographic and clinical characteristics of TB patients

To determine the numbers of different subsets of monocytes/macrophages, 25 patients with new onset TPE and 17 HC were recruited. Their demographic and clinical characteristics are shown in Table 1. There was no significant difference in the distribution of age and gender between the patients and HC. There was no difference in the numbers of PB WBC and neutrophils. The numbers of PB monocytes in the patients were significantly greater while PB lymphocytes were lower than those in the HC (p<0.05 for both). Furthermore, patients had abnormal levels of PF LDH and ADA, but not CEA. In addition, the numbers of PB ESAT-6 and CFP-10-specific IFN-γ secreting T cells and the values of ESR in the patients were significantly higher than that in the HC, demonstrating systemic inflammation in the patients.

**Table 1:** The demographic and clinical characteristics of subjects.

Characteristics	TB (n = 25)	HC (n = 17)
Male/female	13/12	9/8
Age (years)	45 (23–67)	48 (28–68)
Blood		
WBC (×10^9^/l)	5.56 (4.1–13.45)	5.88 (4.35–8.2)
Monocytes (×10^9^/l)	0.58 (0.23–1.09) *	0.44 (0.2–0.62)
Neutrophils (×10^9^/l)	3.91 (2.6–10.66)	3.8 (1.86–6.07)
Lymphocytes (×10^9^/l)	1.17 (1.03–2.1) *	1.62 (0.88–2.08)
Pleural fluid		
WBC (×10^9^/l)	2.34 (0.65–5.12)	-
Mononuclear cells (%)	80 (60–90)	-
CEA (ng/ml)	1.69 (0.46–5.8)	-
DH (U/L)	375 (192–1249)	-
ADA (U/L)	52.6 (3.5–113.2)	-
ESR (mm/h)	53 (15–135) *	5 (3–21)
ESAT-6-specific IFN-γ secreting T cells (SFCs/2.5×10^5^PBMC)	10 (3–50) *	0 (0–3)
CFP-10-specific IFN-γ secreting T cells (SFCs/2.5×10^5^PBMC)	12 (4–50) *	0 (0–4)

Data shown are median (range) of each group of subjects. WBC: White blood cell; CEA: Carcino-embryonic antigen; LDH: Lactate dehydrogenase; ADA: Adenosine aminhydrolase. *p<0.05 vs. the HC.

### Increased numbers of M1-like macrophages in the TPE patients

**Figure 1:**
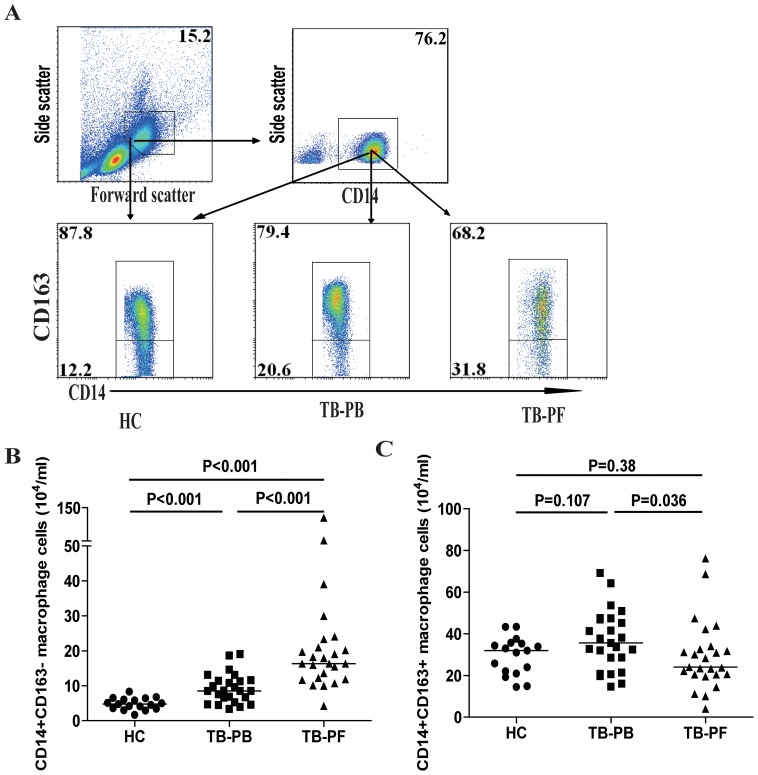
Flow cytometry analyses of macrophages. Peripheral blood mononuclear cells (PBMCs) and pleural fluid mononuclear cells (PFMCs) were obtained from individual subjects and stained with PerCP-anti-CD14 and Alexaflour-conjugated-anti-CD163 or isotype-matched IgG. The cells were gated initially on living mononuclear cells (top left) and then on CD14^+^ macrophages (top right). Subsequently, the numbers of CD14^+^CD163^+^ M2-like and CD14^+^CD163^−^ M1-like macrophages were determined by flow cytometry, based on total numbers of monocytes/macrophages. Data shown are representative charts of flow cytometry and expressed as the mean values of individual subjects (n = 17 for the HC, n = 25 for the patients). The horizontal lines indicate the median values for individual groups.

Next, we characterized the numbers of PB and PF CD14^+^CD163^−^ or CD14^+^CD163^+^ “macrophage-like” monocytes in individual subjects by flow cytometry (Fig. 1). The numbers of PB CD14^+^CD163^−^ M1-like cells in the patients were significantly greater than those in the HC (p<0.001), but less than those in the PF of the patients (p<0.001). In contrast, there was no significant difference in the numbers of PB M2-like cells between the patients and HC. However, the numbers of PB M2-like cells were significantly greater than those in the PF of the patients (p = 0.036). As a result, the ratios of PB CD14^+^CD163^−^ M1-like cells to CD14^+^CD163^+^ M2-like cells in the TPE patients were significantly higher than those in the HC (median:0.24 vs. 0.16, P<0.001), but lower than those in the PF of the patients (0.71 vs. 0.16, P<0.001). Therefore, increased numbers of CD14^+^CD163^−^ M1-like cells existed in the patients with new onset MPE.

### M2-like macrophage-related receptor expression in the TPE patients

**Figure 2:**
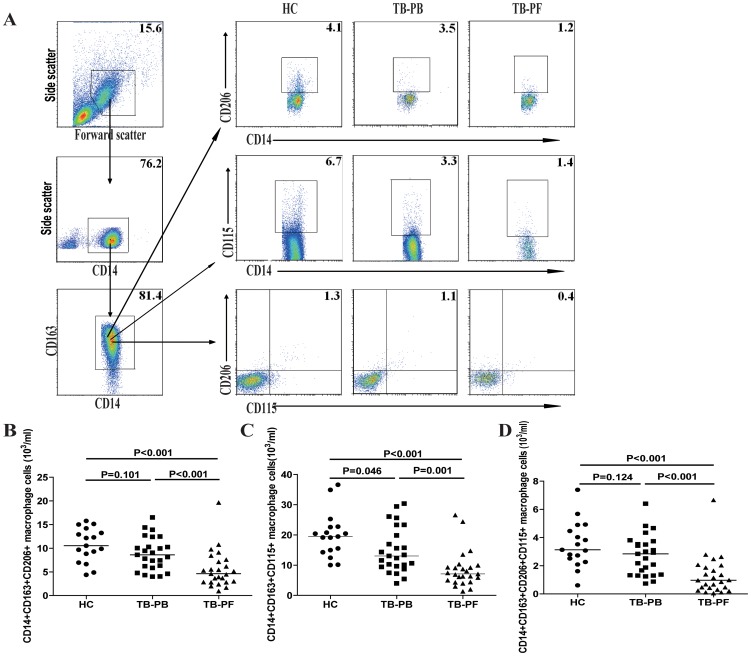
Analysis of PB and PF CD206+ and CD115+ M2-like macrophages. PBMCs and PFMCs were isolated from individual subjects and stained with Alexaflour-anti-CD163, PerCP-anti-CD14, FITC-conjugated anti-CD206, and PE-conjugated anti-CD115 for 30 min. The numbers of PB and PF CD14+CD163+CD206+, CD14+CD163+CD115+, and CD14+CD163+CD206+CD115+ M2-like cells were analyzed by flow cytometry, based on total numbers of monocytes/macrophages. Data shown are representative FACS charts and expressed as the mean numbers of each type of cells per ml of PB and PF of individual subjects from two separate experiments. The horizontal lines indicate the median values for each group.

The mannose receptor (CD206) and macrophage colony-stimulating factor receptor (CD115) are highly expressed by M2 macrophages [14]. Given limited M1 macrophage-specific markers, we next determined the numbers of CD206+ and CD115+ M2-like macrophages by flow cytometry. As shown in Figure 2, the numbers of PB CD14^+^CD163^+^CD115^+^ M2-like macrophages in the TPE patients were significantly less than those in the HC (p = 0.046), and the numbers of PB CD14^+^CD163^+^CD206^+^ and CD14^+^CD163^+^CD206^+^CD115^+^ M2-like cells were slightly less than those in the HC. Furthermore, the numbers of different types of M2-like cells in the PB were significantly greater than those in the PF of MPE patients (p = 0.001, p<0.001, p<0.001, respectively). Therefore, significantly reduced numbers of anti-inflammatory M2-like cells existed in the TPE patients, especially at the pathological site.

### Higher level of IL-12^+^ M1 macrophages and lower level of IL-10^+^ M2 macrophages in patient with new TPE

**Figure 3:**
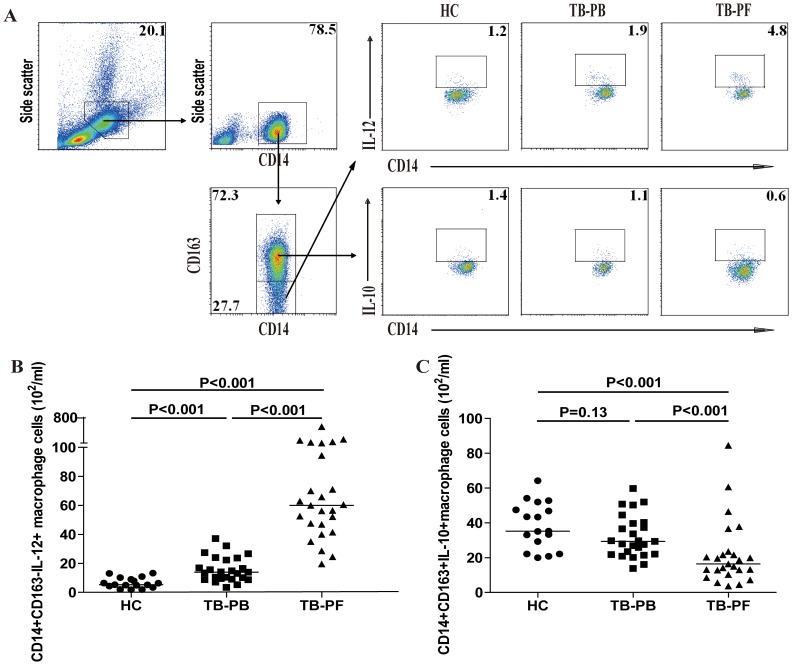
Analysis of PB and PF IL-12^+^ M1 and IL-10^+^ M2 macrophages. PBMCs and PFMCs were isolated from individual subjects and stimulated with LPS/PMA/ionomycin *in vitro*. The cells were stained with Alexaflour-conjugated-anti-CD163, and PerCP-anti-CD14 for 30 min, fixed, and permeabilized, followed by intracellular staining with FITC-anti-IL-10 or PE-anti-IL-12. The numbers of PB and PF CD14^+^CD163^−^IL-12^+^ M1 and CD14^+^CD163^+^IL-10^+^ M2 macrophages in individual subjects were determined by flow cytometry, based on total numbers of monocytes/macrophages. Data shown are representative FACS charts or the mean numbers of each type of cells per ml of PB and PF in individual subjects from two separate experiments. The horizontal lines indicate the median values for each group.

M1 macrophages are characterized by their capability to secrete pro-inflammatory IL-12 whereas M2 macrophages are able to produce IL-10 [18]–[21]. Because there were almost no detectable IL-10^+^ M1-like and IL-12^+^ M2-like cells, we defined the IL-12^+^ M1-like cells as M1 and IL-10^+^ M2-like cells as M2. We examined the numbers of IL-12^+^ M1 and IL-10^+^ M2 macrophages in those subjects by flow cytometry (Fig. 3A). The numbers of PB IL-12^+^ M1 macrophages in the patients were significantly greater than those in the HC (p<0.001), but less than those in the PF of TPE patients (p<0.001, Fig. 3B). In contrast, there was no significant difference in the numbers of PB IL-10^+^ M2 macrophages between the TPE patients and HC. However, the numbers of PB IL-10^+^ M2 cells were significantly greater than those in the PF of TPE patients (p<0.001, Fig. 3C). As a result, the ratios of PF IL-12+ M1 to IL-10+ M2 were significantly greater than that in the PB of patients (3.04 vs. 0.45, p<0.001). Similarly, the ratios of PB IL-12+ M1 to IL-10+ M2 in the patients were greater than that in the HC (0.45 vs. 0.18, p<0.001). These data clearly indicate significantly greater numbers of IL-12^+^ M1, but fewer numbers of IL-10^+^ M2 macrophages in the pathological site of TPE patients, which may be associated with the development of TPE in this population.

### Cytokine responses in patients with new TPE

**Figure 4:**
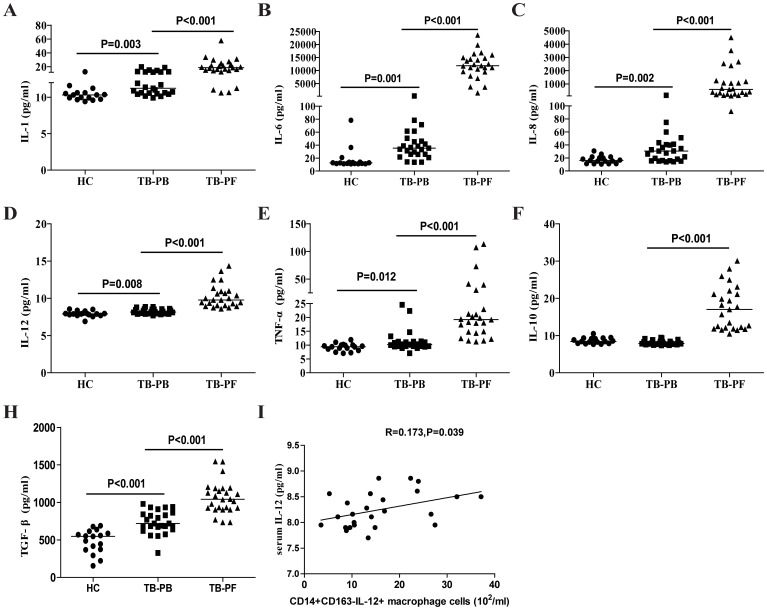
Measurements of serum and PF cytokines in individual subjects. The concentrations of PF and serum IL-1, IL-6, IL-8, IL-10, IL-12, TNF-α, IL-10, and TGF-β1 in individual subjects were determined by CBA/ELISA (A-G). Data shown are the mean values of individual subjects from three separate experiments. The horizontal lines indicate the median values of the different groups; (I): The levels of serum IL-12 are correlated positively with the numbers of PB CD14^+^CD163^−^IL-12^+^ M1 cells in the patients.

Finally, we examined the concentrations of serum and PF cytokines in those subjects by CBA and ELISA. As shown in Figure 4, the concentrations of serum IL-1, IL-6, IL-8, IL-12, TNFα, and TGF-β in the TPE patients were significantly higher than that in the HC (p<0.05 for all), but lower than that in the PF of TPE patients (p<0.05 for all). In contrast, there was no significant difference in the levels of serum IL-10 between the HC and TPE patients, but the levels of serum IL-10 were significantly lower than that in the PF of TPE patients (p<0.001). Further analysis revealed that the concentrations of serum IL-12 were correlated positively with the numbers of PB IL-12^+^ M1 in the TPE patients (R = 0.173, p = 0.039, Fig. 4I). These data clearly indicated higher levels of inflammatory cytokines in the TPE patients.

### The relationship between the levels of cytokines and the values of clinical measures in the TPE patients

**Figure 5:**
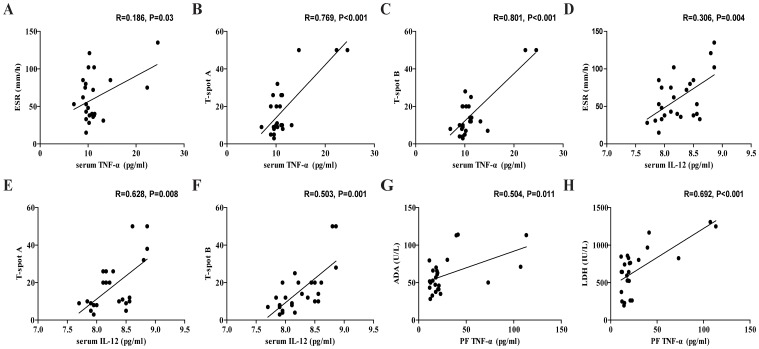
The correlation between the levels of cytokines and the values of clinical parameters in the TPE patients. The potential associations between the concentrations of PF and serum IL-12 or TNF-α and the values of ESR, the numbers of PB ESAT-6-specific IFN-γ-secreting T cells, and CFP-10-specific IFN-γ-secreting T cells, ADA and LDH in those patients were analyzed by the Spearman rank correlation test. Data shown are the values of individual patients (n = 25).

To understand the importance of cytokine responses, we analyzed the potential association of the levels of cytokines with the values of clinical parameters in those patients. We found that the levels of serum TNF-α and IL-12 was correlated positively with the values of ESR (R = 0.186, p = 0.03, Fig. 5A; R = 0.306, p = 0.004, Fig. 5D) and the numbers of ESAT-6- (R = 0.769, p<0.001, Fig. 5B; R = 0.628, p = 0.008, Fig. 5E) and CFP-10-specific IFN-γ secreting T cells in those patients (R = 0.801, p<0.001, Fig. 5C; R = 0.503, p = 0.001, Fig. 5F). In addition, the levels of PF TNF-α were correlated positively with the concentrations of ADA (R = 0.504, p = 0.011, Fig. 5G) and LDH (R = 0.692, p<0.001, Fig. 5H) in the TPE patients. However, there was no significant association of the levels of other cytokines with any of the clinical measures tested in the patients (data not shown). These data suggest that the concentrations of PF and serum M1/M2 cytokines may be valuable markers for evaluating the early process of TPE in humans.

## Discussion

Macrophages are the first line of defense against invading pathogens and act as effectors of the innate immune response [22]–[23]. In this study, we found significantly increased numbers of CD14^+^CD163^−^ M1-like in PB and PF, but decreased numbers of CD14^+^CD163^+^ M2-like macrophages in PF of TPE patients, related to those in the HC. Furthermore, we observed significantly increased numbers of CD14^+^CD163^−^IL-12^+^ M1 macrophages, particularly in the PF, but decreased numbers of CD14^+^CD163^+^IL-10^+^ M2 macrophages in the PF of TPE patients. Similarly, we found that significantly reduced numbers of CD14^+^CD163^+^CD206+, CD14^+^CD163^+^CD115+, and CD14^+^CD163^+^CD206+CD115+ M2-like cells in the PF of TPE patients. In addition, we detected significantly higher levels of serum and PF IL-1, IL-6, IL-8, IL-12, TNFα, and TGF-β1 as well as higher levels of PF IL-10 in the TPE patients. These data clearly indicated that *M.* tb infection induced a polarized pro-inflammatory M1 response. Previous studies have revealed that *M*. tb infection induced pro-inflammatory Th1 and Th17 responses as well as NK cell activation [1], [3], [24]–[33]. Indeed, we detected a higher numbers of antigen-specific T cells in the TPE patients. Given that M1 macrophages promote inflammatory Th1 and Th17 responses and activated NK cells, the increased M1 responses may contribute to the pathogenesis of TPE in humans. Conceivably, detection of abnormally higher levels of M1 responses may help in the diagnosis of TPE.

Interestingly, we detected significantly greater numbers of M1 macrophages and less numbers of M2 macrophages in the PF, as compared with that in the PB in the patients. Similarly, we detected significantly higher levels of cytokines in the PF than that in the PB in the TPE patients. The significantly changed numbers of macrophages and the elevated levels of cytokines in the PF may stem from a strongly inflammatory environment, which preferably activate macrophages towards M1 direction or recruit M1 macrophages. Indeed, high levels of IFN-γ produced by antigen-activated Th1 and activated NK cells were detected in the PF, and IFN-γ is a powerful inducer of M1 cell differentiation and activation [34]. Furthermore, high levels of MCP-1, IL-6, and IL-1 were detected in the PF of TPE patients, and these chemokines are potent for chemoattractants to M1 macrophages. Alternatively, M1 may survive longer than M2 cells in a strongly inflammatory environment [35]. Therefore, further investigation of the molecular mechanisms by which M1 cells accumulate in the PF may reveal new targets for the design of new therapies for patients with TPE.

More importantly, we found that the levels of serum IL-12 and TNF-α were positively correlated with the values of ESR and the numbers of ESAT-6- and CFP-10-specific IFN-γ secreting T cells in the TPE patients, further confirming that increased numbers of M1 cells induced Th1 responses in TPE patients [36]–[37]. In addition, the levels of PF TNF-α were correlated positively with the concentrations of ADA and LDH in the patients. Given that the levels of ADA and LDH are correlated with the pathogenic changes [14]–[17], the significant correlation between the levels of PF TNF-α and ADA or LDH further supports the notion that TNF-α is a major pathogenic factor for tissue damages during the process of TPE. Interestingly, we detected significantly higher levels of PF anti-inflammatory IL-10, but significantly less numbers of PF M2 cells in the TPE patients. IL-10 can be secreted by many types of cells, such as Th2 and Foxp3^+^ Tregs as well as other epithelial cells. A recent study revealed that *M*. tb infection also induced Treg responses at the early process of TB pathogenesis [38] and that vaccination with Bacillus Calmette–Guérin (BCG) also increased the numbers of Tregs in animals and humans [39]. It is possible that the high levels of PF IL-10 may come from infiltrated Tregs, which feedback down-regulate inflammation to minimize the tissue damage. We are interested in further investigating TPE-related immunoregulation in the PF.

Macrophages are importantly inflammatory elements in the pleural effusions. Previous studies have revealed that M2 macrophages represent a major component in malignant pleural effusion (MPE). These M2 macrophages have been suggested to functionally support the growth of tumors by inhibiting anti-tumor T cell immunity [40]. In this study, we found that *M*. tb infection stimulated M1 responses, contributing to the development of MPE. The distribution of different types of macrophages in the PE may serve as a marker for distinguishing TPE from MPE. We are interested in further validating their diagnostic values of different types of macrophages in the PF.

In summary, our data showed that significantly increased numbers of PB and PF M1 cells and decreased numbers of PF M2 cells in the patients with TPE, accompanied by significantly elevated levels of cytokines. To the best of our knowledge, this was the first study on the numbers of different phenotypes of macrophages in patients with new onset TPE. Our findings support the notion that *M*. tb infection induces pro-inflammatory M1 responses, which in turn induce Th1 and Th17 responses, associated with the early process of TPE. The significantly increased M1 response in the PF may serve as a new biomarker for the diagnosis of TPE and to distinguish TPE from other diseases. We recognized that our study had limitations, including a small sample size and the lack of functional studies of different subsets of macrophages as well as without detailed numbers of different types of inflammatory cells in the PF. Thus, further validation of these findings in a bigger population is warranted.
